# Supplementary material for: Band insulator to Mott insulator transition in 1T-TaS2
Source: Nat Commun. 2020 Aug 24;11:4215. doi: 10.1038/s41467-020-18040-4 (PMC7445232; doi:10.1038/s41467-020-18040-4)
Supplement: Supplementary file 1 — Supplementary Information [file 41467_2020_18040_MOESM1_ESM.pdf]

Supplementary Information

Band insulator to Mott insulator transition in 1*T*-TaS_2

Wang *et al*.

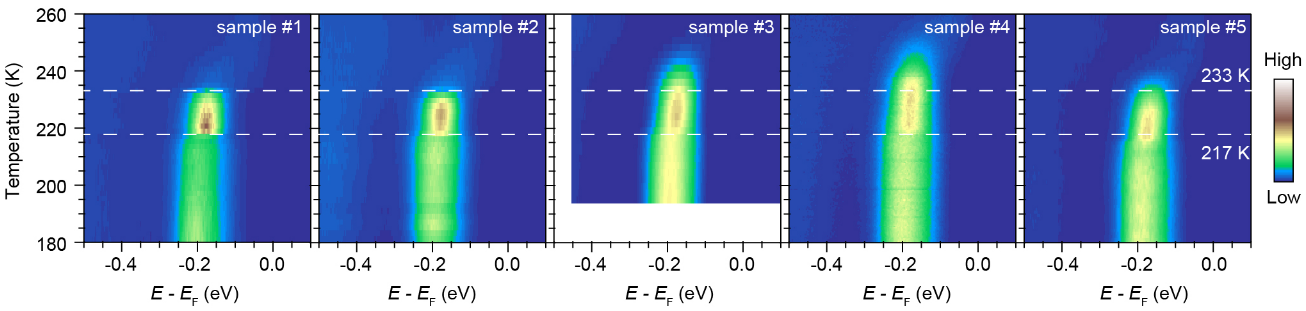

Supplementary Figure 1 | Temperature dependent ARPES data taken on different samples. The energy distribution curves (EDCs) taken at different temperatures are merged into images to better illustrate the phase transitions. The data taken on sample #1 is used in the main text. All data were taken upon heating. The data declare the repetitiveness of our observation well. While dashed lines illustrate the two phase transitions. The intermediate (I) state and the two phase transitions could be observed in all measured samples.

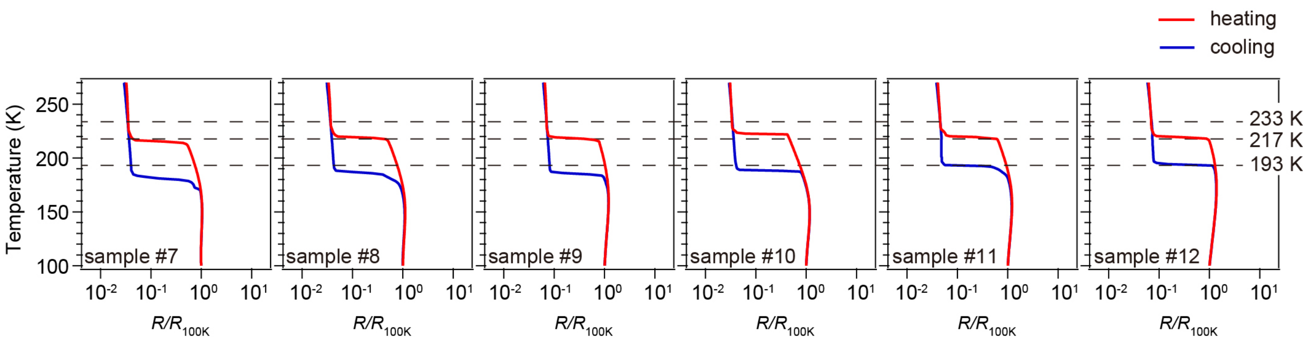

Supplementary Figure 2 | Temperature dependent Resistivity data taken on different samples. The resistivity data were measured in Physical Property Measurement System (PPMS, Quantum Design, Inc.) utilizing the standard four-probe method. The heating and cooling rates are 3 K min^{-1}. The data taken on sample #7 is used in the main text. The dashed lines illustrate the phase transitions at 193, 217, and 233 K determined by ARPES.

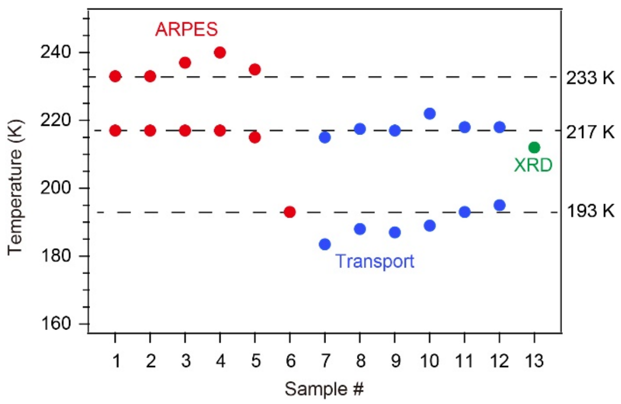

Supplementary Figure 3 | Transition temperatures determined by ARPES, resistivity and X-ray diffraction (XRD) measurements. Three transitions are illustrated using dashed lines. The transition at 193 K is the C-CDW phase transition determined by both ARPES and resistivity data upon cooling. The transition at 217 K is the C-CDW phase transition determined by both ARPES and resistivity data upon heating. The 233 K transition is the I phase transition observed by ARPES upon heating. For technique reasons, we cannot measure the same sample using different techniques. Therefore, there is a small variation of the transition temperatures among different measurements. The standard deviation of transition temperature is less than 10 K, which can be explained by a small sample inhomogeneity. It should be note that such small variation of transition temperatures and sample inhomogeneity cannot explain the observations of the I phase and the 233 K phase transition by ARPES.

Experimental details of the heating processes					
	Sample #1	Sample #2	Sample #3	Sample #4	Sample #5
T_0	80 K, cleave	80 K	80K	80 K	20 K, cleave
R_1	1.5 K min^{-1}	5 K min^{-1}	5 K min^{-1}	5 K min^{-1}	1.5 K min^{-1}
T_1	190 K	160 K, cleave	160 K, cleave	160 K, cleave	160 K
R_2	0.23 K min^{-1}	0.22 K min^{-1}	0.23 K min^{-1}	0.25 K min^{-1}	0.4 K min^{-1}
T_2	300 K	290 K	275 K	295 K	300 K

Supplementary Table 1 | Experimental details of the ARPES measurements for a heating process. The samples were cooled down to T_0 with a rapid fall of temperature (about 20 K min^{-1}) from the room temperature naturally. After adequate cooling for about 10 min at the T_0, the samples were heated to T_1 with a rate (R_1) about 1.5 ~ 5 K min^{-1}. The samples were then heated up slowly to T_2 with a rate (R_2 = 0.22 ~ 0.4 K min^{-1}) to witness the bands evolution near the phase transitions. ARPES data were collected during the heating process. The sample #1 and #5 were cleaved at T_0. The sample #2, #3 and #4 were cleaved at T_1.

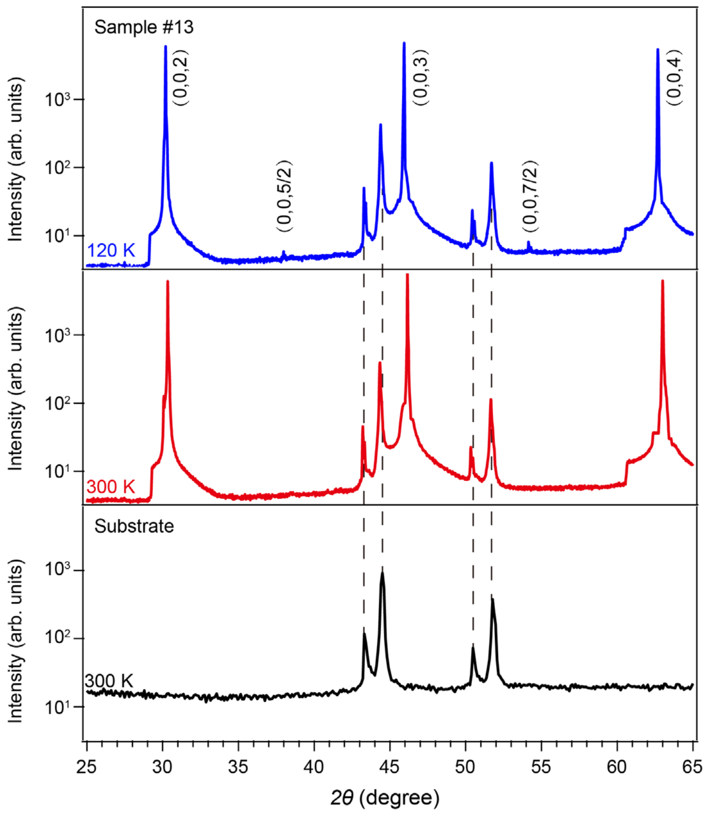

Supplementary Figure 4 | Two theta scans taken on sample #13 and substrate. The data were recorded on a Bruker D8 diffractometer using Cu $K\alpha$ radiation ($\lambda = 1.5418$ Å). The data are shown in log scale to better illustrate the weak (0, 0, *L*/2) peaks. All other diffraction peaks except the (0, 0, *L*) and (0, 0, *L*/2) peaks are from the polycrystalline substrate.

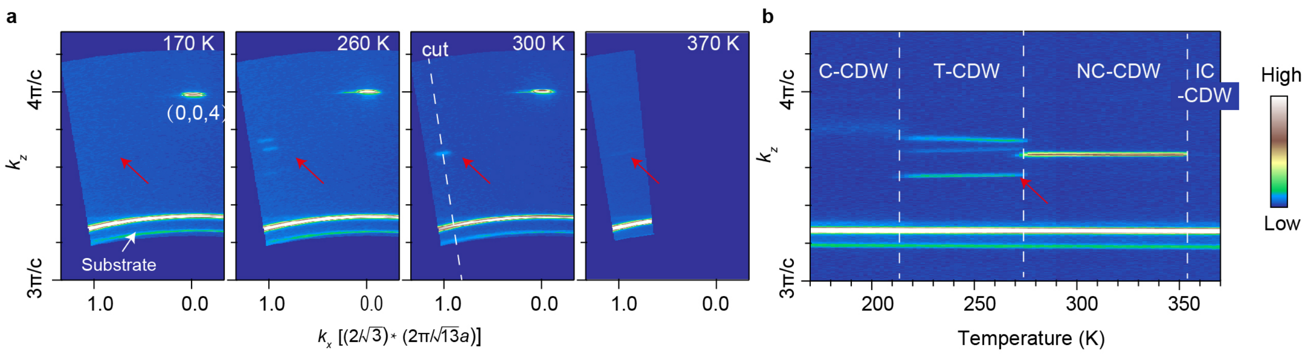

Supplementary Figure 5 | Temperature dependence of the $\sqrt{13}\times\sqrt{13}$ CDW diffraction peak. a, Reciprocal space mappings taken in the k_x-k_z plane, which is ~13.9 degree off from the crystal (1, 0, 0) plane. The data were collected in the C-CDW (170 K), T-CDW (260 K), NC-CDW (300 K), and IC-CDW (370 K) phases during a heating process. **b**, Temperature dependence of the cut taken across the $\sqrt{13}\times\sqrt{13}$ CDW diffraction peak. The CDW diffraction peak emerges in the NC-CDW phase. Its location is consistent with previous studies[1]. In the T-CDW phase, the CDW diffraction peak splits into three peaks, which is consistent with the triple-Q phase reported by Tanda *et al*[2]. In the C-CDW phase below 212 K, the CDW diffraction peak broadens, which could be explained by an increment of stacking randomness along *c* direction.

.

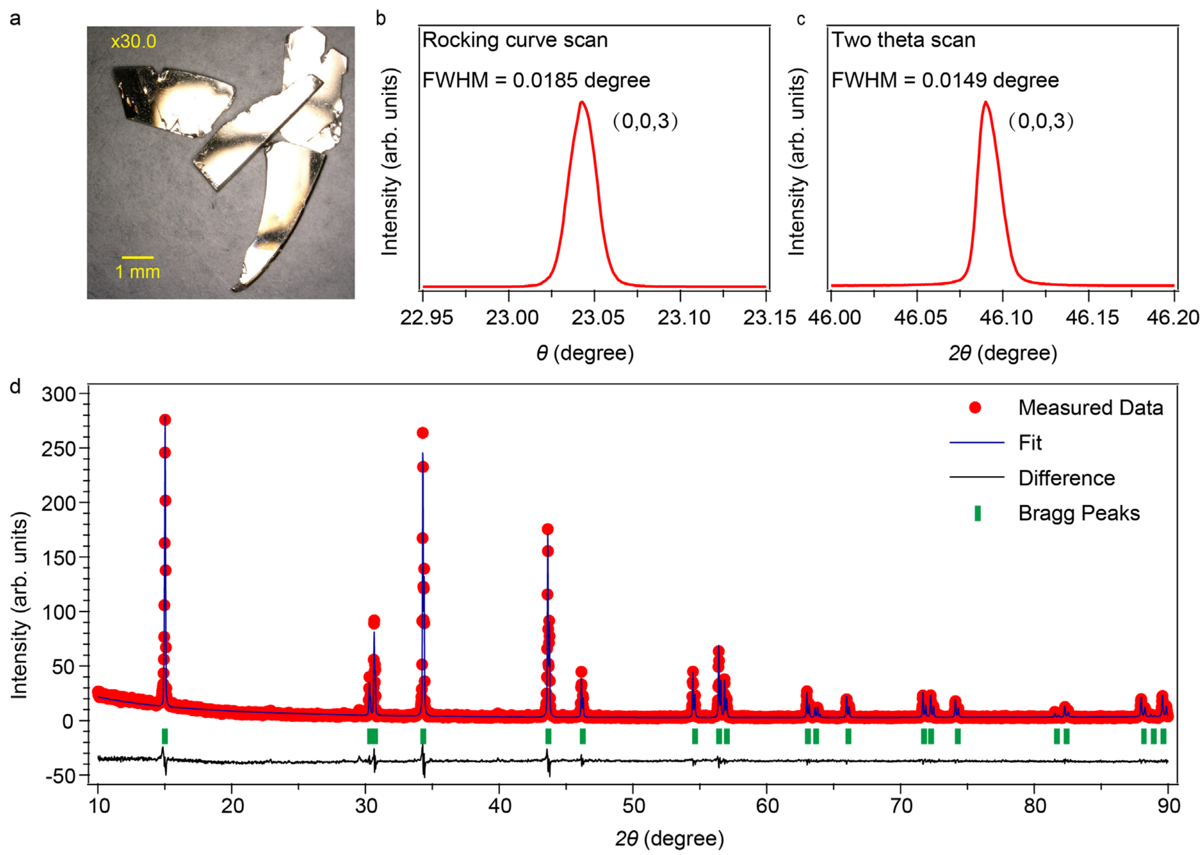

Supplementary Figure 6 | High quality of our 1*T*-TaS_2 samples. a, Large 1*T*-TaS_2 single crystals with mirror-like cleaved surfaces and natural hexagonal edges. **b,** and **c,** Rocking curve and two theta scans taken at 300K using high-resolution X-ray diffractometer (Bruker XRD D8 Discover). The full-width-half-maximums (FWHMs) of the diffraction peaks are around 0.0185° and 0.0149° respectively, indicating the high quality of our sample. **d,** Rietveld refinement of the powder XRD data. The refinement confirms the phase purity of our sample and the trigonal crystal structure of 1*T*-TaS_2 with the *P-3m1* space group with the lattice parameters a = b = 3.366 Å, c = 5.898 Å. These data are well consistent with the powder XRD data reported by Kratochvilova, M. *et al* [3].

Supplementary References

1. Spijkerman, A., de Boer, J. L., Meetsma, A. ,Wiegers, G. A. & van Smaalen, S. X-ray crystal-structure refinement of the nearly commensurate phase of 1*T*-TaS_2 in (3+2)-dimensional superspace. *Phys. Rev. B* **56**, 13757-13767 (1997).
2. Tanda, S., Sambongi, T., Tani, T., Tanaka, S. X-Ray Study of Charge Density Wave Structure in 1*T*-TaS_2. *J. Phys. Soc. Jpn.* **53**, 476-479 (1984).
3. Kratochvilova, M. *et al*. The low-temperature highly correlated quantum phase in the charge-density-wave 1*T*-TaS_2 compound. *Npj Quantum Mater.* **2**, 42 (2017).
